# Impact of malaria diagnostic refresher training programme on competencies and skills in malaria diagnosis among medical laboratory professionals: evidence from Ghana 2015–2019

**DOI:** 10.1186/s12936-021-03796-x

**Published:** 2021-06-08

**Authors:** Mary Tetteh, Duah Dwomoh, Alexander Asamoah, Edward King Kupeh, Keziah Malm, Justice Nonvignon

**Affiliations:** 1 Department of Health Policy, Planning and Management, School of Public Health, University of Ghana, Legon, Accra, Ghana; 2 Ghana Health Service, District Hospital, Begoro, Ghana; 3 Department of Biostatistics, School of Public Health, University of Ghana, Legon, Accra, Ghana; 4 Ghana National Malaria Control Programme, Ghana Health Service, Accra, Ghana; 5 Ghana Health Service, Tema Polyclinic, Tema, Ghana; 6 Health Economics, Systems and Policy Research Group, University of Ghana, Accra, Ghana

**Keywords:** Malaria, Microscopy, Malaria Diagnostic Refresher Training, Medical Laboratory Professionals, Ghana

## Abstract

**Background:**

The quality of malaria test results is crucial for optimal patient treatment and care. The Ghana Health Service is successfully shifting from presumptive clinical diagnosis and treatment of malaria to the Test, Treat and Track (T3) initiative. In line with the initiative, the National Malaria Control Programme (NMCP) set out to improve the capacity of medical laboratory professionals in Ghana through a five-day Malaria Diagnostic Refresher Training (MDRT) to build competencies and skills in malaria diagnosis, especially in the three components of microscopy: parasite detection, species identification and parasite quantification. This study evaluates the impact of the training on malaria microscopy.

**Methods:**

The training which was based on the World Health Organization basic malaria microscopy training guide employed presentations and practical approaches to malaria diagnosis. A total number of 765 medical laboratory professionals from various health facilities across the country were trained every other year from 2015 to 2019 and were included in this evaluation. Evaluation of this training was done using pre-test and post-test microscopy scores. The Negative Binomial fixed effect model was used in determining the overall effect of the training in improving the competencies of the participants on malaria microscopy.

**Results:**

The ability of the medical laboratory professionals to correctly detect malaria parasites increased significantly from a median score of 64% prior to the training to 87% after the training (p < 0.001). The competencies of the medical laboratory scientists to correctly identify malaria parasite species and quantify the number of malaria parasites increased significantly from a median score of 17% and 20% pre-test to 78% and 50% post-test, respectively (p < 0.001). The results showed that participants’ competency level and skill to perform malaria microscopy (species identification, parasite quantification and detection of malaria parasites) increased by approximately two folds after the training compared to the no-training scenario (adjusted rate ratio = 2.07, 95% CI 2.01–2.13, p < 0.001).

**Conclusion:**

The MDRT programme significantly improved participants’ performance of malaria microscopy over a short period of time.

## Background

Though significant efforts have been made in an attempt to reduce burden, malaria continues to be a global health burden with an estimated 229 million cases and 409,000 deaths reported worldwide in 2019, with the hardest hit regions being the World Health Organization (WHO) African Region, which accounted for 94% of the global malaria cases and 94% of the global malaria deaths [1]. Malaria remains endemic in Ghana and is known to be the leading cause of morbidity and mortality especially in children under 5 years of age; it accounts for 2% of the global burden and 3% of deaths globally [1]. Approximately 34% of outpatient department (OPD) cases were reported to be due to suspected malaria in 2017, leading to about 19% of total admissions (out of which 55.6% were children under 5 years) and 2% of total deaths (54.6% in children under 5 years) [2].

As part of measures to reduce the malaria morbidity and mortality burden, Ghana rolled onto the test, treat and track (T3) initiative of the WHO in 2010 [3]. According to the T3 initiative, presumptive treatment of malaria using only clinical signs is reduced to the barest minimum and every suspected case must be routinely confirmed using the malaria rapid diagnostic test kit (RDT) or microscopy [4]. The RDTs are a relatively simple and quick way of diagnosing malaria and are usually used in areas with less skilled labour and no electricity, but it has disadvantages of being expensive: with a cost of up to 0.6 USD per test for the Histidine Rich Protein 2 (HRP2) only test kits and up to 0.9 USD per test for the *Plasmodium* lactate dehydrogenase (pLDH) test kits while microscopy costs up to 0.5 USD per slide [5]; unable to detect very low parasitaemia with a parasite detection threshold of 100–200 parasites per microlitre of blood; and not suitable for drug efficacy monitoring [6]. On the other hand, microscopy is the gold standard in malaria diagnosis as it is able to detect low parasitaemia threshold of 100 parasites per microlitre of blood in the thin film and about 50 parasites per microlitre of blood in the thick film [5]; identify and describe species and stages, quantify parasite density and is also used for treatment response monitoring. The sensitivity and specificity of this diagnostic tool is however of great concern as skilled laboratory professionals trained in microscopy are needed to give reliable results [7]. Accurate diagnosis of malaria is important in reducing misdiagnosis of the disease and helps in identifying other fatal non-malarial causes of infection, reducing wastage of and possible resistance to antimalarial drugs and enables the effective management of febrile patients [8]. A false positive malaria report will result in clinically similar diseases being treated as malaria, reducing specificity, promoting over-use of anti-malarial drugs and reducing the quality of care for patients [9] whereas a false negative malaria report will result in withheld treatment which can cause disease progression from uncomplicated malaria to severe malaria with its consequence of death. Additionally, *Plasmodium ovale* can form latent intrahepatic hypnozoites that may cause disease several months or even years after the primary infection [10] if missed and the additional Primaquine medication, which is to treat the liver stage is not administered. The quality of microscopy varies from one laboratory to another mainly due to the lack of quality supplies and reagents used in the various laboratory settings; and the techniques employed.

Lack of training and limited supervision has led to poor characterization of the various *Plasmodium* species with the exception of *Plasmodium falciparum*, inaccurate estimation of the parasite density; and the non-detection of mixed infections and low parasitaemia [11]. While some studies have reported that some health workers resist most negative laboratory results mainly due to mistrust in the performed tests, other publications have questioned the competencies and skill of malaria microscopists with respect to the accuracy, quality, and an overall lack of strict standards [12–14]. One of the most important requirements in the development and maintenance of effective microscopy especially in malaria endemic regions is well trained laboratory professionals who are able to accurately prepare and read blood slides [15]. The National Malaria Control Programme in Ghana aims at improving access to quality malaria diagnosis for all suspected cases at all levels by building the needed capacity for malaria diagnosis and enforcing adherence to the test, treat and track policy guidelines [2]. In order to build capacity and provide quality malaria diagnosis, the Malaria Diagnostic Refresher Training was organized with the objective of equipping laboratory professionals across the country with the essential knowledge, skills and competences in malaria diagnosis especially in malaria microscopy. This 5-day intensive practical training session pays attention to rapid diagnostic testing as a simulation exercise and emphasizes more on preparation and staining of blood smears; and practical microscopy sessions made up of parasite detection, species identification and parasite quantification. The Malaria Diagnostic Refresher Training (MDRT) has been conducted for the past 10 years in Ghana but information on the immediate impact of the training in imparting knowledge and improving the skill of the participants in malaria diagnosis especially microscopy is scanty. The evaluation of the MDRT will give an indication of its impact in building the capacity of laboratory professionals in malaria diagnosis to prevent misdiagnosis of malaria. This study, therefore seeks to assess the impact of the MDRT on malaria diagnosis.

## Methods

### Study design

This study was a retrospective interventional study where the pre-test and post- test microscopy results of all the participants who took part in the malaria diagnostic refresher training for 2015, 2017 and 2019 were collected and analysed.

### Study sites

The training was conducted in all the ten regions of Ghana. Each region is made up of districts with private and public health facilities such as Regional (secondary) and District (primary) hospitals; Health centres and Polyclinics as well as Mission hospitals, Quasi-government facilities and Private hospitals and laboratories. Participants were selected from these facilities to participate in the Malaria Diagnostic Refresher Training.

### Intervention

#### Approach

The training consisted of a five-day residential intensive session, which was based on the WHO basic malaria microscopy learner’s guide [16]. The training was held through theoretical presentations and practical sections in preparing of blood film and performing RDTs.

#### Study participants

The trainees were medical laboratory professionals belonging to the grade of either medical laboratory scientists (MLS), Technical Officers (TO) or medical laboratory assistants (MLA). They were selected by their respective Regional medical laboratory scientists based on their training needs. The number of participants per training session was a minimum of 20 to a maximum of 30 laboratory professionals per region.

#### Facilitators for the training

Facilitators on the other hand consisted of WHO certified malaria microscopists (Level 1 and 2), Nationally certified malaria microscopists (NCAMM level A and B), Regional malaria supervisors as well as National malaria supervisors from the National Malaria Control Programme and Clinical Laboratory Unit of the Ghana Health Service.

The National competency assessment for malaria microscopy (NCAMM) programme is organized in accordance with WHO standards using the WHO Quality Assurance (QA) manual version 2 [17] as a guide. The composition of the slides used for this assessment is similar to what is used in the WHO external competency assessment. Additionally, blood films prepared and stained by the participants are assessed. Both the slides examined and the blood films prepared are graded as outlined in the WHO QA manual.

#### Content of the training workshop

The training was made up of theoretical presentation sessions and practical sessions as outlined in the timetable (Table 1). The timetable for the MDRT (Table 1), which was developed using the WHO basic malaria microscopy guide, gives an outline of all the day to day activities carried out in the training.

**Table 1:** Timetable used in the malaria diagnostic refresher training

Time	Monday	Tuesday	Wednesday	Thursday	Friday
8:00–8:20	Welcome Address	Recap 1 and Presentation of Results	Recap 2 and Presentation of Results	Recap 3 and Presentation of Results	Recap 4
8:20–9:00	Introduction, ground rules, expectations, groups formation etc	Examination of Blood films	Review of Post-test 1 microscopy slides	Review of Post-test 2 microscopy slides	Review of stained Blood films
9:00–9:40	Pre-test (Preparation of blood films)	MDRT Preparation of blood films (5 slides)	MDRT Preparation of blood films (5 slides)	MDRT Preparation of blood films (10 slides) (5-Positive and 5 Negative)	Laboratory safety in malaria diagnosis
9:40–10:10	Conduct Pre-test (theory)	Quantification of malaria parasites	Artefacts, pseudo-parasites, mixed infections & other parasites (theory)		Introduction to G6PD Testing
10:10–10:40	Use, care, and storage of the microscope	Review of Pre-test microscopy slides	Collection of blood for malaria tests	Parasitological stains (theory)	Malaria situation in Ghana Update
10:40–11:00	Snack Break	Snack Break	Snack Break	Snack Break	Snack Break
11:00–12:40	Conduct Pre-test-Practical (6 slides–session 1)	Microscopy Post-test Assessment 1 (6 slides–session 1)	Microscopy Post-test Assessment 2 (6 slides–session 1)	Microscopy Post-test Assessment 3 (6 slides–session 1)	Malaria DHIMS Data from participating facilities Development of SOPS Conduct Post-test (theory) Evaluation
12:40–13:30	Pre-test (Staining of blood films)	MDRT Staining of blood films (5 slides)	MDRT Staining of blood films (5 slides)	MDRT Staining of blood films (5 slides)	Recap 5 and presentation of MDRT final results
13:30–14:30	Lunch	Lunch	Lunch	Lunch	Lunch
14:30–16:00	Conduct Pre-test-Practical (6 slides–session 2)	Microscopy Post-test Assessment 1 (6 slides–session 2)	Microscopy Post-test Assessment 2 (6 slides–session 2)	Microscopy Post-test Assessment 3 (6 slides–session 2)	DISCUSSION–NEXT STEPS –Report writing, requisition of logistics, lobbying etc
16:00–16:30	Preparation and staining of thick and thin Blood films	Parasite Life Cycle	Principles and procedures of malaria RDTs	Malaria quality assurance-MICROSCOPY and RDT	Closing remarks
16:30 – 18:00	Conduct Pre-test-Practical (6 slides–session 3)	Microscopy post-test Assessment 1 (6 slides-section 3)	Microscopy post-test Assessment 2 (6 slides section 3)	Microscopy Post-test Assessment 3 (6 slides–session 3)	Snack Break
18:10–18:30	Snack Break	Snack Break	Snack Break	Snack Break	Snack Break
18:30–20:30	Facilitators’ Examination and Assessment of Participants’ Stained Blood Films and Meeting				

#### Practical sections

For the practical sessions on blood film preparation and staining, each participant was provided with 10 frosted-end microscope slides where they used one slide as a spreader to prepare 9 thick and thin blood films on same slide and selected the best 5 films for staining and assessment. The trainees volunteered for their blood samples to be collected as part of practical blood collection training while permission was sought from the head of laboratory of the nearest health facility to obtain blood infected with malaria parasite and it was granted. All other supplies for blood collection and quality malaria blood film preparation and staining were provided to participants as part of the supplies arrangements for the training.

For the practical session on malaria RDTs, the participants were paired and provided with RDT kits and supplies for testing. The RDT tests were conducted by all participants on their respective pairs and also assessed as part of the simulation exercise. The malaria positive blood sample was also tested using the RDT to demonstrate positivity. Facilitators assessed the tests by marking the theory with the right answers, assessing prepared and stained blood films against the WHO criteria for assessing blood films as outlined in the WHO QA manual and scored. The thick film was measured with a ruler to assess if it was 12 mm in diameter and whether news print can be read under it before staining; after staining, it was observed to ascertain if 90% of the smear was intact. The distance between the frosted end and the thick film as well as that between the thick and thin film was examined to check if it was 10 mm; the thin film was assessed for a distinct head, body and tail. Microscopically, the thin film was assessed for the presence of a monolayer of red blood cells, while the thick film was examined for completely lysed red blood cells; finally, the staining was examined by assessing the colour of the white blood cells and red blood cells in the thin and thick film respectively. This was to establish if the staining clearly distinguishes the various stages of the parasite if present and the white blood cells from the background. The microscopy examinations in terms of parasite detection, species identification and parasite quantification were scored using a computerized excel database.

### Equipment and supplies

Each participant was provided with a light microscope, Immersion oil, two tally counters and a calculator for the examination of validated slides. The validated slides for practical microscopy parasite detection, species identification and parasite quantification were obtained from the National Archive for Malaria Slide Bank in Ghana composed mainly of the three species *P. falciparum, P. ovale,* and *Plasmodium malariae* as well as low density (169–277 parasites per microlitre of blood), medium density (1789–2921parasites per microlitre of blood) and high-density (90,103–233,234 parasites per microlitre of blood). The slide bank is composed of over 6000 slides of different species which were internationally validated according to WHO protocols in developing a malaria slide bank as outlined in the WHO QA manual. Universal Power Saver (UPS) devices were also connected in place to avoid power disruptions during power outages before power generator plants at training venues were turned on.

### Training sessions

The morning session involved preparation of blood films, theoretical presentations and a review of the previous days’ microscopy assessment whiles the afternoon session entailed practical microscopy examinations (post-test assessments) staining of prepared blood films and another theoretical presentation. Each new day began with a presentation of the results and an open and intensive review of the slides examined the previous day where participants were freely allowed to share their opinions.

There was pre-test on practical preparation of blood films, followed by a theory pre-test consisting of questions on malaria parasitological diagnosis and pathogenesis. The participants were then taken through a presentation on use, care and storage of the microscope to introduce amateur trainees to the microscope to avoid any misuse and damage; after which they then took a practical malaria microscopy pre-test where they examined a set of 18 slides comprising three *P. falciparum,* two *P. malariae*, two *P. ovale*, one mixed infection of *P. falciparum* and *P. malariae* and another one with *P. falciparum* and *P. ovale,* one low density, two medium density, one high density and five negative slides with time limit of 10 min per slide using the light microscope. This was to measure their capacity in parasite detection, species identification and parasite quantification.

The blood films that were initially prepared were later stained. The objective of conducting these pre-tests was to assess the baseline level of each participants’ theoretical knowledge and practical skills in blood film preparation and staining; and in malaria microscopy.

The facilitators used the criteria for assessing blood films as outlined in the WHO QA manual to assess the blood films, and graded them according to the prescription in the manual. The results of the slide examinations were scored at the end of each day’s activities and activities for the next day was then planned. This presentation paved way for the practical review of the pre-test validated slides. In this session, there was an open presentation of the performance of each trainee with respect to the previous days’ slide examination prior to the review of the slides. This was to allow the participants to share their opinions and recognize some of the mistakes they made during the slide examinations; it was also to identify gaps in the trainees’ microscopy skills and competencies. Afterwards, the slides were made available according to their compositions for participants to examine according to their needs and to fill the gaps identified with the guidance of the certified malaria microscopist.

On the final day of the training the pre-test and post-test results for the theoretical assessments, blood films and microscopy examinations for each trainee was displayed and each trainee presented with a certificate of participation.

The microscopy assessments were conducted under examination conditions where no talking or sharing was allowed and each participant was given 10 min to examine each slide for parasites and species identification or parasite quantification. Different sets of slides with similar composition were used for each of the tests. There were separate pretest slides for parasite detection, species identification and parasite quantification.

### Mode of assessment of effect of the intervention

A pre-test was conducted before the start of the training and a post-test after the training. Effectiveness was assessed by a difference in the performance of participants in these two tests.

The trainees’ microscopy examinations were scored and graded using a standardized computer-based template; each score was presented over a hundred percent (100%). The emphasis of this study was on malaria parasite detection, species identification and parasite quantification among the laboratory professionals over a five-year period from 2015 to 2019.

The trend differences in the outcome of the training in relation to some of the demographic characteristics of the participants such as cadre, previous in-service training experience, sex and facility type were assessed in a univariate analysis whiles the Negative Binomial and Poisson fixed effect models were used in finding the net effect of the training on the performance of the participants.

### Outcome measures

The outcome of the trainee results was set premised the WHO grading system.

*Parasite detection*: this is the ability of the laboratory professional to indicate whether a malaria parasite is truly present (sensitivity) or truly not seen (specificity). Each microscopist must obtain an 80% or more score in sensitivity and specificity to be deemed competent in parasite detection.

*Species identification*: even though there are five types of *Plasmodium* species infecting humans worldwide, there are only three found in Ghana: *P. falciparum, P. ovale* and *P. malariae*. The participants are to know and differentiate between the four developmental stages of each *Plasmodium* species, namely the ring, trophozoite, schizont and gametocyte. The participants must also be able to differentiate between the parasites using their morphological features. A participant must score 80% or more in species identification.

*Parasite quantification*: the detected malaria parasites must now be quantified. This is done in the thick film by counting the number of asexual stages of the malaria parasites per field, while counting white blood cells alongside until a total of a minimum of 200 WBCs are counted. In the thin film, parasite quantification is enumerated by counting the number of infected red blood cells alongside a total minimum of 5000 RBCs. Trainees must attain a score of 40% or more out of the total number of slides examined to be deemed quantifying accurately. Each of the internationally validated slide falls within a certain predetermined range; the trainees are expected to achieve a count of ± 25% of the count range of each slide as determined by the international validators.

### Thick film using WBCs

$$\begin{aligned} \text{Parasite Density} =\, & \frac{\text{Number of parasites counted}}{\rm {Number\,of\,WBCs\,counted}} \times 8000\, \text{(standard no. of WBCs per ul)}\\ =\, & \text{Parasites per ul of blood} \end{aligned}$$

### Thin film using RBC

$$\begin{aligned}\text{Parasite Density} =\, & \frac{\text{Number of parasitized RBCs counted}}{\rm {Number\,of\,total\,RBCs\,(parasitized\,and\,non-parasitized)} \, }\times 5000000 \text{ RBCs per ul}\\ =\, & \text{Parasites per ul of blood} \end{aligned}$$

### Statistical analysis

The pre and post-test microscopy results of all the participants who took part in the MDRT for 2015, 2017 and 2019 were retrieved and entered into a Microsoft Excel 2016 spread sheet and exported to Stata IC version 16 (Stata Corp, College Station, Texas, USA). Descriptive statistics (i.e., frequencies and percentages) were used to quantify categorical variables. The median and interquartile ranges were used to summarize the pre- and post-test scores since the scores were heavily skewed (non-normal distribution). The Wilcoxon matched-pairs signed-rank test was used to compare the differences in median scores before and after the training. The Poisson and Negative Binomial fixed effect models with robust standard error were used in determining the net effect of the training on the knowledge score of the participants. The likelihood ratio test was used to test for overdispersion (conditional variance exceeds the conditional mean) in the count outcomes (discrete scores obtained by each participant). However, for the purposes of comparing effect size estimates, rate ratios from both models were reported. Sub-group analysis of the impact estimate was conducted to ascertain how the impact estimate varies across the years of implementation of the intervention, administrative regions, the different cadre of laboratory professionals as well as different health facilities. All statistical analyses were conducted using Stata 16 (Stata Corp, College Station, Texas, USA) and p-values less than 0.05 were considered statistically significant.

## Results

### Demographic characteristics of study participants

A total of 765 laboratory professionals from the 10 regions of the country comprising 613 males and 152 females of different cadres were enrolled in the study as shown in Table 2. Out of this number, 122 (15.9%, Table 2) have participated in a similar malaria microscopy training previously before taking part in the current one while 643 (84.1%; Table 2) have not participated in any similar malaria microscopy training prior to this training experience.

**Table 2:** Demographic characteristics of study participants

Variable	Frequency	Percentage
Region		
Ashanti	92	12.0
Brong Ahafo	82	10.7
Central	74	9.7
Eastern	78	10.2
Greater Accra	94	12.3
Northern	67	8.8
Upper East	66	8.6
Upper West	64	8.4
Volta	79	10.3
Western	69	9.0
Sex		
Male	613	80.1
Female	152	19.9
Training history		
Had prior training	122	15.9
No prior trained	643	84.1
Facility type		
Clinic	33	4.31
Health centre	118	15.42
Hospital	544	71.1
Polyclinic	50	6.54
Laboratory	20	2.61
Facility ownership		
CHAG	142	18.56
Government	480	62.75
Private facility	108	14.12
Quasi Government	35	4.58
Year of training		
2015	243	31.8
2017	244	31.9
2019	278	36.3
Cadre of staff trained		
Laboratory assistant	109	20.9
Technical officer	195	37.4
Medical laboratory scientist	218	41.8

### Effect of MDRT on the performance of malaria microscopy among study participants

There was an overall improvement in the percentage median score for the participants after they had taken part in the training. Before the training was carried out, the median percentage score of the ability of the participants to correctly detect the presence or absence of a malaria parasite was 64%, however, the median percentage score increased to 87% after the training (p < 0.001; Table 3). The overall knowledge gained in malaria microscopy for participants increased from 32.7% before the training to 71.3% after the training and was statistically significant (p-value < 0.001; Table 3).

**Table 3:** The effect of the malaria diagnostic refresher training on participants performance in malaria microscopy

Variable	Pre-test	Post-test	P-value
	Median (iqr)%		
Detection of malaria parasites	64 (18)	87 (14)	< 0.001***
Species identification of malaria parasites	17 (22)	78 (29)	< 0.001***
Quantification of malaria parasites	20 (25)	50 (50)	< 0.001***
Overall malaria microscopy performance	32.7 (17.3)	71.3 (20.7)	< 0.001***

iqr: interquartile range

*p < 0.05, **p < 0.01, ***p < 0.001

### The effect of the malaria diagnostic refresher training on malaria microscopy by characteristics of study participants

The results from the Wilcoxon matched-pairs signed-rank test showed that individuals who had ever received a similar training (pre-test 42.3%, post-test 75.7%; Table 4) had higher pre-test and post-test median percentage scores compared to individuals who had never had the training before (pre-test 31%, post-test 71%; Table 4). When the results were stratified by cadre, it was observed that the medical laboratory scientists (pre-test 35.3%, post-test 72.3%; Table 4) had the highest pre and post-test median scores, followed by the technical officers (pre-test 32.3%, post-test 69%; Table 4) with the laboratory assistants (pre-test 27.3%, post-test 63.7%; Table 4) recording the lowest pre and post-test median scores. However, there was no statistically significant difference in the pre and post-test median scores with respect to gender (Table 4).

**Table 4:** The effect of the malaria diagnostic refresher training on malaria microscopy by characteristics of study participants

Variable	Pre-test detection	Post-test detection	Pre-test speciation	Post-test speciation	Pre-test quantification	Post-test quantification	Pre-test overall	Post-test overall	P-value
	Median (Interquartile range) %								
Regions									
Ashanti	55 (26)	90 (21)	11 (22)	83 (27.5)	0 (25)	40 (30)	26.7(15.8)	70 (23)	< 0.001
Brong Ahafo	64 (23)	86 (21)	17 (22)	78 (22)	0 (25)	75 (50)	29.3 (17)	72.7 (21)	< 0.001
Central	64 (16)	91 (20)	17 (22)	71 (32)	22.5 (25)	50 (50)	35.5 (13)	69.3 (23)	< 0.001
Eastern	70 (23)	87 (20)	22 (20)	80 (30)	25 (40)	50 (47)	38.3 (18)	69.5 (18.7)	< 0.001
Greater Accra	70 (22)	86 (20)	22 (22)	83 (22)	22.5 (40)	50 (50)	34.5(16.7)	71.8 (17.3)	< 0.001
Northern	64 (23)	86 (14)	17 (22)	75(32)	0 (25)	50 (50)	32.3(15.7)	69 (22.7)	< 0.001
Upper East	64 (23)	91 (14)	14 (22)	72.5 (39)	0 (25)	50 (50)	31.3 (16)	72.7 (30)	< 0.001
Upper West	64 (23)	82 (20)	33 (37)	78 (29)	25 (25)	50 (50)	40.3(22.2)	72.2 (20.5)	< 0.001
Volta	64 (18)	82 (20)	14 (33)	78 (31)	20 (25)	50 (55)	34.3(17.7)	71.3 (25.7)	< 0.001
Western	61 (21)	90 (13)	14 (33)	86 (33)	0 (25)	40 (30)	30 (14.7)	71 (18.3)	< 0.001
Sex									
Male	64 (23)	86 (14)	17 (22)	78 (26)	20 (25)	50 (50)	33 (17.3)	71.3 (21)	< 0.001
Female	64 (23)	90 (14)	17 (22)	78 (32)	0 (25)	50 (50)	32.3(19.5)	70 (21.5)	< 0.001
Trained history									
Had prior training	71 (29)	90.5 (20)	33 (42)	86 (33)	25 (40)	50 (50)	42.3 (25)	75.7 (19)	< 0.001
No prior training	64 (23)	86 (14)	17 (22)	78 (32)	0 (25)	50 (50)	31 (15.3)	71 (20.3)	< 0.001
Type of facility									
Clinic	64 (16)	86 (14)	33 (19)	83 (23)	0 (25)	50 (50)	34.6 (14)	71.6 (24.3)	< 0.001
Health centre	64 (23)	84 (20)	17 (22)	67 (39)	0 (25)	50 (50)	30 (19.3)	66.5 (23.6)	< 0.001
Hospital	64(18)	90 (14)	17 (22)	78 (22)	20 (25)	50 (50)	32.7 (17.3)	71.3 (21.3)	< 0.001
Laboratory	64 (22.5)	85 (20)	22 (32)	68.5 (34)	0 (25)	50 (50)	32.7(22.5)	71.3 (17.2)	< 0.001
Polyclinic	65.5 (20)	90 (18)	22 (22)	88 (33)	25 (40)	60 (50)	37 (18)	74.8 (17.3)	< 0.001
Facility ownership									
CHAG	64 (18)	90 (21)	22 (22)	83 (23)	20 (25)	50 (50)	32.5 (14.3)	70.7 (18.3)	< 0.001
Government	64 (18)	87 (14)	17 (22)	78 (26)	20 (25)	50 (50)	33.3 (17.8)	72 (21.8)	< 0.001
Private	64 (23)	86 (20)	17 (22)	71 (30)	0 (25)	50 (50)	30 (18.2)	68.8 (20.7)	< 0.001
Quasi Government	55 (21)	82 (36)	17 (22)	71 (30)	20 (25)	50 (50)	32.3 (21)	72.7 (28)	< 0.001
Year of training									
2015	64 (25)	90 (11)	22 (27)	78 (26)	20 (25)	60 (55)	34 (19)	73.3 (21)	< 0.001
2017	64 (18)	90.5 (18)	17 (33)	71 (32.5)	25 (25)	50 (50)	32.8 (17)	70.5 (21.7)	< 0.001
2019	64 (21)	86 (14)	22 (22)	88.5 (33)	0 (25)	25 (25)	32.3 (16)	69 (19.7)	< 0.001
Cadre of participants trained									
MLS	64 (23)	91 (20)	17 (22)	83 (23)	25 (25)	50 (50)	35.3 (17)	72.3 (21.3)	< 0.001
TO	64 (23)	86 (14)	17 (22)	78 (32)	0 (25)	50 (50)	32.3 (17)	69 (22)	< 0.001
LA	57 (20)	86 (20)	14 (29)	71 (39)	0 (25)	25 (25)	27.3 (15)	63.7 (21)	< 0.001

MLS: Medical laboratory scientists, TO: Technical officers, MLA: Medical laboratory assistants (MLA)

### The impact of MDRT on malaria microscopy competency among the study participants

The training increased the rate of malaria parasite detection score by 1.36 times (95% CI 1.33–1.39, p < 0.001; Table 5) after training compared to before training. Malaria species identification and parasite quantification scores increased by approximately fourfold; (adjusted rate ratio; aRR = 3.99, 95% CI 3.67–4.34; p < 0.001) and (aRR = 3.4, 95% CI 3.01–3.88; p < 0.001; Table 5), respectively. The participants competency level to perform malaria microscopy (species identification, parasite quantification and detection of malaria parasite) increased by approximately two folds after the training [aRR = 2.07, 95% CI 2.01–2.13, p < 0.001; Table 5).

**Table 5:** Impact of MDRT on malaria parasite detection, species identification and parasite quantification among study participants

Variable	Poisson regression model with robust standard error	Negative binomial regression model with robust standard error
	RR [95% CI]	RR [95% CI]
Malaria parasite detection		
Before intervention	Ref	Ref
After intervention	1.34 [1.32–1.35] ***	1.36 [1.33—1.39]***
Malaria species identification		
Before Intervention	Ref	Ref
After Intervention	3.31 [3.26–3.37] ***	3.99 [3.67–4.34]***
Malaria parasite quantification		
Before intervention	Ref	Ref
After intervention	2.92 [2.86–2.98] ***	3.41 [3.01–3.88]***
Overall performance of participants		
Before intervention	Ref	Ref
After intervention	2.03 [2.00–2.06] ***	2.07 [2.01–2.13]***

RR: rate ratio, Ref: Reference category

***p < 0.001

## Discussion

This study estimated the impact of MDRT on malaria microscopy among the trained medical laboratory professionals from selected public and private health facilities from all the ten regions of Ghana. This training was organized as part of the efforts by the Ghana National Malaria Control Programme to improve malaria microscopy diagnosis among medical laboratory professionals. The performance of the participants in detecting malaria parasites, malaria species identification and malaria parasite quantification by the count system were assessed using the pre-test and post-test practical microscopy score.

Generally, the refresher training resulted in an improvement in the knowledge, competencies and skill set of the participants in malaria microscopy, which is evident in the post-test performance of the participants. The finding is consistent with those of other studies in Angola [11], Nigeria [18] and Ghana [19]. Another study on microscopy for Acid Fast Bacilli training [20] reported similar finding to the finding that participants who had benefitted from a similar previous training performed better than those who were attending the training for the very first time. However, their study reported that participants with diploma were observed to have performed significantly better in comparison to the degree holders whereas the results of this study reported otherwise; the medical laboratory scientists who are the degree holders performed better than the technical officers who are the diploma holders and the laboratory assistants who are the certificate holders. The observed difference could be due to the total number of slides examined; a total of 10 slides were used in their study whiles our study employed a total of 18 slides. Additionally, their study participants were only diploma and degree holders with the majority (75.3%) being diploma holders while our trainees were certificate, diploma and degree holders. Another comparable study conducted in Nigeria showed no statistically significant differences in the pre-test and post-test median scores among the three categories of participants in practical malaria microscopy [21]. This current study employed the use of Microsoft Corporation power point presentations for the theory sessions and microscopy only for the practical session whereas the study conducted in Nigeria employed the use of videos and pictures in addition to the practical sessions.

Participants from the polyclinics recorded the best performance in both pre-test and post-test scores, followed by those in the clinics, hospitals and private laboratories, with participants from the health centres recording the lowest pre-test and post-test scores. This could be because participants in the polyclinics and hospitals are medical laboratory scientists who frequently perform microscopy and have access to regular in-service training opportunities while members in the health centres are usually technical officers or lab assistants with less microscopy experience because they mostly use the malaria rapid diagnostic test (RDT) in diagnosing malaria; some health centres may even not have microscopes in their laboratories. Participants from the private health facilities may also have recorded low pre-test and post-test performances because most of them are not usually exposed to new trends and guidelines in malaria microscopy; more so, most of them may not have the opportunities to partake in in-service trainings in malaria microscopy. A study in Ethiopia [20] reported that participants from private facilities performed better in the training than those from the government facilities while Lim et al. stated that in a similar refresher training for francophone countries in Africa [22], participants from the central or regional level facilities performed better than those from the peripheral level facilities.

The use of microscopy in diagnosing malaria through parasite detection, species identification and quantification are thought of as the gold standard. Accurate examination of a slide, however, requires highly skilled and competent medical laboratory professionals, especially when parasite densities are low. The analysis of results from this study, showed an improvement in the parasite detection skill of the participants with the difference in the median pre-test and post-test scores being 23%. However, a pre-test median score of 64% showed that most of the laboratory professionals had a degree of parasite detection skills before they took part in the training. Similar studies conducted in other African countries presented similar findings. While the reports of ten-day and seven- day trainings held in Nigeria presented mean pre-test detection scores of 53.9% and 48.9% with mean post-test detection scores of 70.7% and 56.8%, respectively [18, 21], an assessment carried out on five-day malaria diagnostic competency assessment trainings for laboratory professionals in nine African countries showed an improvement of the detection skills of the participants of 19.1 percentage points [23] which is lower than the 23% reported in this study.

Species identification was observed to have had the most impressive improvement margin of 61 percentage points per this assessment. The pre-test median score of 17% was very low compared to 18.9% reported by Aiyenigba, Ojo (18) and 27.9% reported by Olukosi, Agomo (21) whereas the post test score of 78% in this study was higher than the post-test scores of 38.3% and 39.2% reported respectively in the other studies. The *P. falciparum* species is the commonest and most fatal malaria causing species in this country so the other species are seldom seen, therefore, making identification of the morphologic features of the other parasite species quite difficult. Other studies have also revealed that medical laboratory scientist have a difficulty in differentiating malaria species microscopically [24, 25].

Conducting parasite quantification is vital in monitoring disease severity and tracking the efficacy of the anti-malarial drugs especially in severe hospitalized malaria cases. In this study the quantification scores saw an improvement of 30 percentage points (20% pre-test rising to 50% post-test); comparable to the observations made for species identification, parasite quantification scores in the current study were quite low at the beginning of the Malaria Diagnostic Refresher Training for reports of other trainings too. While one study in Nigeria [21] reported of a quantification score which improved from 0 to 25%, another Nigerian study [18] reported of an increase from 4.2% to 27.9% with [23] reporting an average increase of 37.9 percentage points from 15.5% pre-test score to 53.3% post-test score. Interestingly, another study conducted in Francophone African countries [22] reported that the quantification test scores increased from 0 to 33% for basic training, but when participants from the basic training were enrolled into the advanced training, the participants recorded an improvement percentage margin of 38 percentage points where the pre-test was 25%, but increased to 69% post-test. These findings, therefore, suggest that the parasite counting skill can be acquired by the laboratory professionals through short-term trainings and continuous practice.

The differences observed in the pre-test and post-test scores of the participants could perhaps be due to the fact that the first basic skill in malaria microscopy is detection of the malaria parasite hence the ability of most participants to accurately report the presence or absence of a parasite; however, species identification and parasite quantification using the count method was introduced later by the WHO [26]. With most health care workers especially, laboratory professionals and prescribers being used to the plus system (a semi quantitative way of grading parasites within a particular range) of quantifying malaria parasites, the laboratory professionals lacked the skills involved in accurately identifying and quantifying the species using the count method (expressing the quantity of parasites against the number of WBCs or the quantity of parasitized RBCs against RBCs) whereas the prescribers had difficulty in interpreting the parasitaemia results.

Despite the findings of this study and their importance in informing policies regarding training of laboratory professionals for management of other communicable diseases, there are limitations, which are worth noting. Firstly, the data used for this evaluation did not capture variables such as age of participants, the number of working years and number of years with experience in malaria microscopy which could affect the performance of the participants and confound the training outcomes. Secondly, information on the cadre for the 2015 training group was not available however all other needed data including the pre and post-test scores were available for usage in this analysis. Therefore, the cadre analysis carried out in this study used only 2017 and 2019 data. Finally, only the immediate outcome of the training is being assessed, the medium to long-term impact of the training at the facility level is not being assessed in this study.

## Conclusion

The Malaria Diagnostic Refresher Training has been shown to greatly improve malaria microscopy outcomes of the participants within the five-day period of intensive training with respect to parasite detection, species identification and parasite quantification skills. Therefore, periodic malaria microscopy in-service training and perhaps a review of the malaria diagnosis content for training of medical laboratory professionals to include pre-service training as well as regular on-site training and supervision by the already trained professionals may be necessary to assist in producing highly skilled and competent medical laboratory professionals who can accurately and reliably diagnose malaria microscopically.

## Data Availability

All data generated and analysed are included in this article. The raw data is available with the corresponding author upon reasonable request.

## Abbreviations

CHAG: Christian Health Association of Ghana
MDRT: Malaria Diagnostic Refresher Training
NMCP: National Malaria Control Programme
RDT: Rapid Diagnostic Test
SOP: Standard Operating Procedure
WHO: World Health Organization
